# Additive Value of B-Type Natriuretic Peptide on Rest ^201^Tl-Dipyridamole Stress ^99m^Tc-Sestamibi Gated Myocardial SPECT in Patients with Normal Left Ventricular Systolic Function

**DOI:** 10.4061/2010/642045

**Published:** 2010-04-12

**Authors:** Jung-Ju Sir, Young-Seok Cho, Woo-Young Chung, Bon-Kwon Koo, In-Ho Chae, Dong-Ju Choi, Hyo-Soo Kim, Byung-Hee Oh, Young-Bae Park

**Affiliations:** ^1^Department of Internal Medicine, Inje University College of Medicine, Busan 614-735, South Korea; ^2^Department of Internal Medicine, Seoul National University College of Medicine, Seoul 156-707, South Korea; ^3^Cardiovascular Center, Seoul National University Bundang Hospital, Seoul 110-799, South Korea; ^4^Seoul National University Boramae Medical Center, Seongnam-si, Gyeonggi-do 463-707, South Korea; ^5^Cardiovascular Center, Seoul National University Boramae Medical Center, Shindaebang 2-Dong 425, Dongjak-Gu, Seoul 110-779, South Korea; ^6^Cardiovascular Center, Seoul National University Hospital, Seoul 110-799, South Korea

## Abstract

We evaluated whether BNP has additive value to SPECT in patients with normal left ventricular (LV) systolic function. Data from 224 consecutive patients who underwent rest ^201^Tl-dipyridamole stress ^99m^Tc-sestamibi gated SPECT and coronary angiography due to chest pain were analyzed. Patients with true positive SPECT showed significant higher BNP level than those with false positive defect (38.5 (19.0–79.8) versus 19.0 (9.3–35.8), *P* = .01). Patients with true negative SPECT also showed significantly lower BNP level than those with false negative SPECT (39.0 (23.0–77.0) versus 22.0 (15.0–43.0), *P* = .002). In multivariate analyses, elevated BNP level (using a cut-off value of 23.0 pg/mL) was the strongest and independent predictor of CAD in overall patients (OR 2.75, 95% CI: 1.50–5.023, *P* = .001) and patients with positive SPECT (OR 3.34, 95% CI: 1.51–7.37, *P* = .003). The area under the receiver-operating characteristic curve for CAD in overall patients and patients with positive SPECT was 0.673 (95% CI: 0.603–0.743, *P* < .001) and 0.694 (95% CI: 0.602–0.786, *P* < .001), respectively. This study suggests that BNP level has additive diagnostic value to SPECT findings in predicting CAD in patients with normal LV systolic function.

## 1. Introduction

Previous studies have reported that B-type natriuretic peptide (BNP), synthesized as pre-proBNP mainly in the ventricular myocardium and secreted in response to ventricular volume and pressure overload, has close relation with angiographic severity and could be a potential biomarker for myocardial ischemia even in patients with preserved LV systolic function [1–4]. In patients with coronary artery disease, myocardial ischemia may cause transient left ventricular (LV) systolic and diastolic dysfunction and consequently increase LV wall stress, leading to elevated plasma natriuretic peptide level or directly promotes the release of natriuretic peptide independent from left ventricular wall stress [5, 6]. Recently, it was discovered that natriuretic peptides are associated with exercise-induced myocardial ischemia in patients with coronary artery disease and BNP level at rest can significantly improve diagnostic power of treadmill test in patients with suspected coronary artery disease and normal left ventricular ejection fraction [7–11]. 

 Stress myocardial perfusion imaging is also widely used noninvasive modality for the diagnosis and risk stratification of patients with suspected myocardial ischemia [12, 13]. The incidence of myocardial perfusion abnormalities at rest or during stress is a hallmark of coronary artery disease [14]. The sensitivity of stress technetium-99m sestamibi single photon emission computed tomography (SPECT) for detecting coronary artery disease has been reported to exceed 90%, the specificity ranges between 53% and 100%. However, the diagnostic accuracy of SPECT is profoundly influenced by the presence of tissue attenuation such as diaphragm or breast [15]. Also, in patients with multivessel coronary artery disease, the perfusion abnormalities may be unremarkable or even missed due to relatively balanced hypoperfusion between the stenosed vessels [16]. Therefore, combining natriuretic peptides as markers for inducible myocardial ischemia may be useful for boosting diagnostic accuracy of SPECT. In this study, we investigated the relationship of BNP level with inducible myocardial ischemia on stress technetium-99m sestamibi myocardial SPECT and evaluated whether combining BNP measurement with SPECT could improve diagnostic accuracy to predict significant coronary artery disease in patients with normal LV systolic function.

## 2. Material and Methods

### 2.1. Patients

This study analyzed patients who underwent rest ^201^Tl/stress ^99m^Tc sestamibi dual-isotope myocardial perfusion SPECT with dipyridamole stress and coronary angiography for evaluation of chest pain at Seoul National University Hospital between December 2005 and February 2007. Patients with atrial fibrillation, significant valvular heart disease, or renal insufficiency (creatinine clearance by Cockcroft and Gault equation ≤25 mL/min or serum creatinine ≥2.3 mg/dL) were excluded to avoid confounding increase in BNP level due to causes other than myocardial ischemia per se. We also excluded patients with evidence of myocardial infarction (pathologic Q waves in the resting electrocardiogram) and fixed perfusion defect on the SPECT to differentiate the patients with “inducible myocardial ischemia” from those with irreversible myocardial injury (scar).

### 2.2. SPECT Image Acquisition and Reconstruction

Rest ^201^Tl-dipyridamole stress ^99m^Tc-sestamibi gated SPECT was performed prior to coronary angiography. First, 111 MBq ^201^Tl was injected and rest SPECT was performed using a dual-head camera (Vertex EPIC; ADAC Laboratories) equipped with a low-energy high-resolution collimator. Dipyridamole (0.56 mg/kg) was then injected over 4 minutes and 925 MBq ^99m^Tc-sestamibi was injected 3 minutes after stress. Gated ^99m^Tc-sestamibi SPECT was performed 90 minutes after stress using the same camera. Thirty-two step-and-shoot images were acquired at intervals of 3° over 25 s per step. For gating, 16 frames per cardiac cycle with a prefixed RR interval and 40% windows were used. ^201^Tl SPECT was reconstructed using a Butterworth filter with a cut-off frequency of 0.35 cycles per pixel and an order of 10 and ^99m^Tc-sestamibi SPECT was reconstructed using a Butterworth filter with a cut-off frequency of 0.45 cycles per pixel and an order of 10.

### 2.3. Interpretation for Perfusion Abnormalities

For regional analysis, the myocardium was divided into 20 segments, and segmental perfusion and thickening were quantified using automatic quantifying software (AutoQUANT; ADAC Laboratories), which awarded a quantitative value and scored the segment according to 4-point scoring system, 0 for normal uptake, 1 for mildly decreased uptake, 2 for moderately decreased uptake and 3 for perfusion defect. The perfusion abnormality at each segment was attributed to each artery territory (left anterior descending, circumflex and right coronary artery). For determining the perfusion abnormalities, we took into consideration not only computer-based interpretation but also physician analysis to avoid the false positive perfusion abnormalities due to attenuation defect especially in women. We used three nuclear variables, summed stress score (SSS), summed rest score (SRS), and summed difference score (SDS). SSS was obtained by calculating the sum of scores of 20 segments of stress ^99m^Tc-sestamibi images. SRS was calculated on a similar basis. Summed difference (reversibility) score (SDS) was determined by the sum of differences between the SSS and SRS in each segment. According to SSS, we considered myocardial ischemia as mild (between 4 and 8), moderate, (9–13) and severe (>13) [17].

### 2.4. Calculation of Ventricular Volumes and Ejection Fraction in SPECT

Short-axis tomographic images were further processed using Cedars quantitative gated SPECT (QGS) software. The LV ejection fraction (LVEF), end-systolic volume (ESV) and end-diastolic volume (EDV) were calculated automatically by Cedars QGS software after making three-dimensional surface images of endocardium and epicardium.

### 2.5. Coronary Angiography

In our study, coronary angiography was considered as the gold standard method of imaging coronary stenoses. All patients underwent coronary angiography by the standard techniques. Intracoronary nitroglycerin (0.1–0.2 mg) was administered into affected coronary artery, just before the acquisition of the coronary angiogram. The degree of stenosis was expressed as percentage of reduction in the internal luminal diameter in relation to normal reference. Patients with >70% diameter stenosis in any of the three major coronary arteries (left anterior descending, circumflex and right coronary artery) or any major large branch with more than 2 mm in diameter were considered to have significant CAD (for left main, ≥50% diameter stenosis).

### 2.6. Echocardiography

An M-mode and 2D transthoracic echocardiography (TTE) was performed in all patients prior to coronary angiography. All patients showed normal LV systolic function (LVEF ≥ 50%) and no regional wall motion abnormalities. Peak early (*E*) and late (*A*) diastolic transmitral Doppler flow velocities were obtained from apical four-chamber view using pulsed wave Doppler, with sample volume positioned at the mitral valve tip.

### 2.7. BNP Assay

All the venous blood samples were collected in EDTA tubes for measurement of BNP levels. Assays for BNP were performed with a Triage B-type Natriuretic Peptide test (Biosite Diagnostics, Inc., San Diego, California) which is fluorescence immunoassay for the quantitative determination of BNP in whole blood and plasma specimens, with a turnaround time of 15 minutes and measurement range of 5–5000 pg/mL [18, 19]. This method correlates the fluorescence measurement to BNP concentration by using an internal calibration curve. In a previous study by Wieczorek et al., the coefficient of variation (CV) for intra-assay precision was 9.5% at 28.8 pg/mL, 12.0% at 584 pg/mL, and 13.9% at 1180 pg/mL. The CV for intrassay precision was 10% at 28.8 pg/mL, 12.8% at 584 pg/mL, and 14.8% at 1180 pg/mL. The CV for interassay variation was 10% at 28.8 pg/mL, 12.4% at 584 pg/mL, and 14.8% at 1180 pg/mL [20].

### 2.8. Statistical Analysis

Continuous variables were presented as mean values ± standard deviations (SD) except BNP data (median with interquartile). BNP was log-transformed for analyses because of its skewed distribution. Within-group comparisons were performed with the Mann-Whitney *U* test or Kruskal-Wallis test. A measure of the linear association between two variables was evaluated using Spearman's rank statistics and all statistical tests were two-tailed. A multivariate regression analysis was performed on variables found to be significant predictors of CAD in univariate analysis. Cut-off values for BNP were determined by performing receiver-operating characteristic (ROC) curve analysis. To evaluate the diagnostic performance of BNP and SPECT, sensitivity, specificity, positive and negative predictive values were calculated. A *P* value of <.05 was considered as significant. The statistical package used was SPSS 13.0 (SPSS Inc. Chicago, Illinois, USA).

## 3. Results

### 3.1. Baseline Characteristics

The baseline characteristics are presented in Table 1. Patients were categorized according to the SPECT results. The patients (*n* = 224) were 62.6 years old, and 60.7% of them were men. Patients with significant coronary artery disease were 121 (54.0%) patients. SPECT was true positive in 94/142 (61.8%) and true negative in 55/82 (67.1%). Patients with true positive SPECT (*n* = 94) were older (*P* = .007) and had a significantly higher prevalence of hypertension (*P* = .03) than those with false positive SPECT (*n* = 48). Patients with true positive SPECT had significantly higher summed stress score and summed difference score than those with false positive SPECT (*P* < .001 for all). In patients with negative SPECT (*n* = 82), patients with false negative SPECT (*n* = 27) had a significantly lower HDL-C level (*P* < .01) and creatinine clearance (*P* = .01) and showed a significantly higher prevalence of diabetes (*P* = .02) and hypertension (*P* = .03) than those with true negative SPECT. Patients with true positive SPECT showed significant higher BNP than those with false positive defect (38.5 (19.0–79.8) versus 19.0 (9.3–35.8), *P* = .01). Patients with true negative SPECT also showed significantly lower BNP than those with false negative SPECT (39.0 (23.0–77.0) versus 22.0 (15.0–43.0), *P* = .002). There was no significant difference in left ventricular ejection fraction, left ventricular/atrial size (LVESV, LVEDV, LA), and several Doppler parameters in echocardiography (*E* wave velocity, *A* wave velocity, *E/A* ratio and deceleration time) between each group.

### 3.2. Correlation between BNP and Clinical Variables, Echocardiographic and SPECT Parameters

The correlations of logarithmically transformed BNP with clinical variables and parameters of echocardiography and SPECT are shown in Table 2. Logarithmically transformed BNP showed a significant correlation with age (*r* = 0.367, *P* < .001) and *A* wave (*r* = 0.194, *P* < .008) and a significant inverse correlation with creatinine clearance (*r* = −0.226, *P* = .001) in overall patients (Table 2). There was also similar correlation between logarithmically transformed BNP and these variables in patients with true positive SPECT (Table 2).

**Figure 1:**
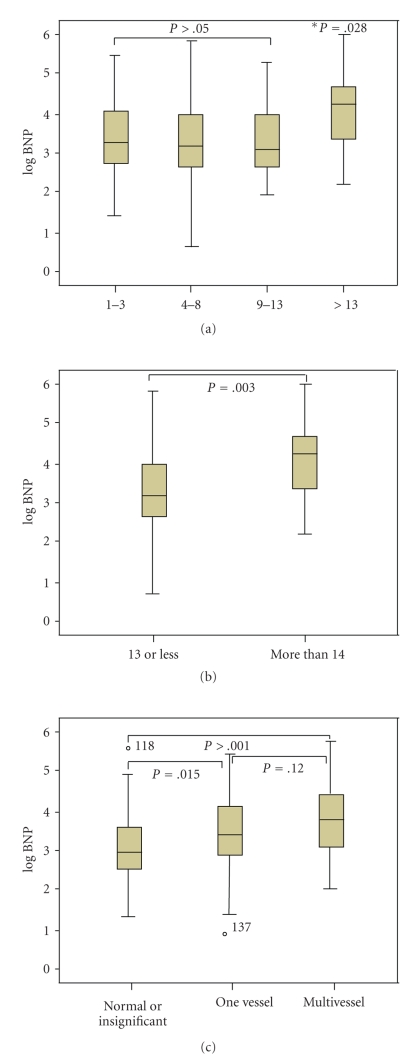
Box plots showing BNP levels (in logarithm) in patients according to the summed stress score (a) and (b) and angiographic severity (c). According to summed stress score, patients were grouped as mild (between 4 and 8), moderate (9–13), and severe (>13). Boxes show median and interquartile ranges and bars represent upper and lower range limits.

Analyzed in terms of the severity or extent of myocardial ischemia detected in SPECT, there was a significant correlation between logarithmically transformed BNP and summed rest score (*perfusion defect at rest*) in patients (*r* = 0.249, *P* < .001) and those with positive SPECT (*r* = 0.309, *P* = .001) (Table 2). Although summed difference score (*reversible ischemia*) also showed a significant correlation with logarithmically transformed BNP in patients with positive SPECT (*r* = 0.172, *P* = .043), the correlation was much weaker than summed rest score. According to summed stress score, patients were divided as mild (between 4 and 8), moderate (9–13), and severe (>13) ischemia. Patients with severe myocardial ischemia (SSS > 13) showed significantly higher BNP level than those with mild or moderate ischemia and there was no significant difference in BNP level between those with mild and moderate ischemia (Figure 1). Similarly, there was also no significant difference in BNP level between those with single vessel disease and multivessel disease in angiography (44.5 (21.8–84.5) versus 31.0 (18.0–65.0) (13.0–37.0), *P* > .05) (Figure 1).

### 3.3. Multivariate Analysis for Predictors of Coronary Artery Disease

In multivariate analyses, elevated BNP level (using a cut-off value of 23.0 pg/mL) was a significant independent predictor of coronary artery disease in overall patients (OR 2.75, 95% CI: 1.50–5.023, *P* = .001) and patients with positive SPECT (OR 3.34, 95% CI: 1.51–7.37, *P* = .003) (Table 3). In patients with negative SPECT, however, BNP level was not an independent predictor for significant CAD (OR 1.93, 95% CI: 0.65–5.74, *P* = .24).

### 3.4. Diagnostic Performance of BNP for Coronary Artery Disease

**Figure 2:**
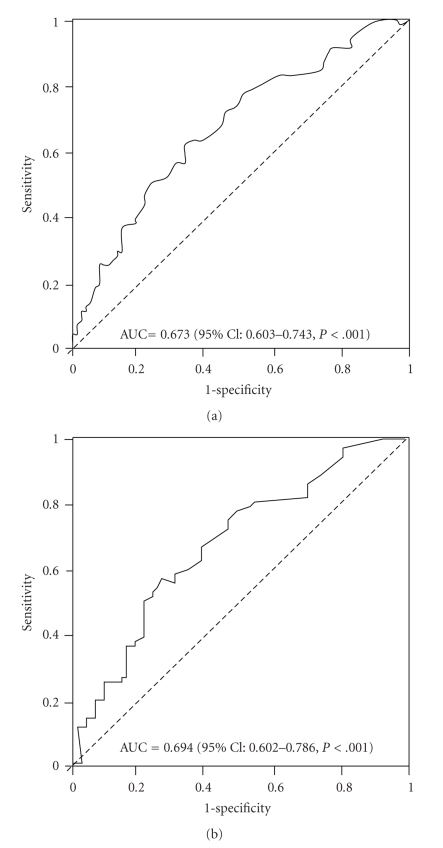
ROC curve of BNP (in logarithm) for predicting CAD in overall patients (a) and patients (b) with positive SPECT. Area under ROC curve is 0.673 and 0.694, respectively.

In overall patients, SPECT showed a sensitivity of 77.7%, a specificity of 41.3%, and positive/negative predictive value of 66.2%/67.1% for detecting coronary artery disease (Table 4). The most sensitive BNP cut-off, identified by receiver-operating characteristic analysis, was 23.0 pg/mL and sensitivity, specificity, positive, and negative predictive value were 70.2%, 56.3%, 65.4%, and 61.7%, respectively. In patients with positive SPECT (*n* = 142), plasma BNP (cut-off value of 23.0 pg/mL) showed a sensitivity of 68.1%, a specificity of 57.1%, and positive/negative predictive value of 78.0%/50.0%. The area under the receiver-operating characteristic curve for significant coronary artery disease in overall patients and patients with positive SPECT was 0.673 (95% CI: 0.603–0.743, *P* < .001) and 0.694 (95% CI: 0.602–0.786, *P* < .001), respectively (Figure 2). 

In terms of territory of ischemia on SPECT, elevated BNP level (≥23.0 pg/mL) predicted significant coronary artery disease on LAD with a sensitivity of 73.7%, a specificity of 57.1%, and positive/negative predictive value of 75.7%/54.5% in patients with perfusion defect on LAD territory. In patients with perfusion defect on RCA or LCx territory, elevated BNP level (≥21.0 pg/mL) predicted significant coronary artery disease on RCA or LCx with a sensitivity of 71.9%, a specificity of 65.4%, and positive/negative predictive value of 82.6%/51.5%.

## 4. Discussion

In the present study, we found that BNP was the strongest and independent predictor of CAD in overall patients with normal left ventricular systolic function even after the adjustment of age and renal dysfunction. Our results coincide with the previous study by Kragelund et al. showing that patients with high NT proBNP level had a significantly higher prevalence of CAD regardless of the left ventricular systolic function [8]. However, our results show that the role of BNP in patients with normal left ventricular systolic function as a diagnostic tool for CAD seems to be limited. BNP (with cut-off value 23 pg/mL) showed only modest sensitivity and specificity for predicting CAD in overall patients and in patients with negative SPECT (Table 4). This modest prediction level of CAD in patients with normal left ventricular systolic function and no RWMA (evidence of infarction on echocardiography) may be explained as follows. First, in our study, only patients with severe abnormal scan (SSS > 13) showed significantly higher BNP level. This suggests that mild to moderate degree of inducible myocardial ischemia may not increase baseline BNP level at rest significantly in patients with normal left ventricular systolic function. Second, this might be attributed to less correlation between BNP at rest and inducible myocardial ischemia in patients without fixed defect on SPECT. There was no significant correlation between BNP and SDS (inducible myocardial ischemia) in overall patients but only a weak correlation in patients with positive SPECT. In this study, SRS (perfusion defect at rest) showed modest but a significant correlation with BNP level in overall patients and patients with positive SPECT. These findings suggest that baseline BNP level *at rest* is associated with a degree of fixed perfusion defect rather than inducible ischemia. 

In several previous studies, there were controversies on relationships between BNP and inducible myocardial ischemia on SPECT. In a previous study by Wong et al., BNP level showed a significant correlation with the degree of reversible ischemia rather than rest score on SPECT in patients who have neither any history of myocardial infarction nor evidence of LV systolic dysfunction on SPECT [21]. This is contrary to our data. In our study, we performed transthoracic echocardiography in all patients and excluded patients if they showed any RWMA on TTE or pathologic Q waves on resting electrocardiogram, and all the patients underwent coronary angiography. After all, we found that there was only a weak correlation between SDS and BNP even in patients with true positive SPECT. Another study by Bibbins-Domingo et al. showed that the relation between elevated BNP level and inducible myocardial ischemia in patients with stable CAD was evident in patients who had a history of myocardial infarction, less than those without history of myocardial infarction [9]. This result accords with our data and suggests that baseline BNP level may have a significant correlation with fixed defect rather than inducible myocardial ischemia on SPECT.

In the previous study, SSS > 13 suggested high risk for cardiac events such as cardiac death and nonfatal myocardial infarction and candidates for cardiac catheterization and revascularization [17]. Therefore, if an angina who has normal left ventricular systolic function and no regional wall motion abnormality (RWMA) on TTE shows elevated resting BNP level (even in normal range) which cannot be explained by any other cause, it may have extensive CAD and may require invasive management. In our study, patients with true positive SPECT were older and had significantly lower creatinine clearance than those with false positive SPECT, but BNP was a significant and independent predictor of CAD after adjustment with these factors (age and renal dysfunction). In patients with negative SPECT, however, BNP was not an independent predictor of CAD. This may be attributed to several reasons. First, we defined “significant CAD” as presence of angiographically critical coronary lesions defined as greater than 70% diameter stenosis. Therefore, patients showing negative SPECT who have intermediate lesion (50%–70% diameter stenosis) might have elevated BNP level as much as patients with more than 70% stenosis. Second, multivessel disease was more frequent among patients with true positive SPECT compared to those with false negative SPECT (59.6% versus 51.9%, resp.) (Table 1). On the other hand, one-vessel disease was more frequent in patients with false negative SPECT than those with true positive SPECT (48.1% versus 40.4%, resp.) (Table 1). The less severe extent of CAD in patients with false negative SPECT may contribute to the insignificant BNP distribution in negative SPECT group. Finally, relatively small number of patients (*n* = 27) with false negative SPECT in our study may limit statistical significance.

This study had several limitations. First, it analyzed the retrospective cohort data from a relatively small number of patients in a single, tertiary referral hospital. Second, we studied a group of consecutive patients who were referred to coronary angiography for clinically suspected ischemic heart disease. Because coronary angiography is often advised in populations with a higher prevalence of risk factors and may not be performed in patients with atypical chest pain and false positive SPECT, the pretest likelihood of CAD was high in this study. So, the high pretest probability in our patients would have contributed to the modest sensitivity of BNP level and SPECT by this selection bias. Third, we defined significant CAD as diameter stenosis >70% and therefore the diagnostic performance needs be reassessed for the intermediate lesions. Finally, as BNP is not specific for myocardial ischemia and known to be associated to age and sex, with higher values in females and older individuals, age and sex-specific cutoff levels should be redefined.

In conclusion, the diagnostic value of BNP for CAD in patients with normal left ventricular systolic function is limited. However, elevated BNP level, a significant and strong predictor of CAD, may suggest extensive CAD in angina patients with positive SPECT.

## Figures and Tables

**Table 1:** Baseline characteristics according to the SPECT results.

	(+) SPECT			(−) SPECT		
	TP	FP	*P*	TN	FN	*P*
Patients, *n* (%)	94 (61.8)	48 (38.2)		55 (67.1)	27 (32.9)	
Gender, *n* (M/F)	59/35	23/25	.09	36/19	18/9	.91
Age, years	64.2 ± 9.0	59.9 ± 9.6	.007**	61.0 ± 10.2	64.9 ± 8.0	.14
BMI, kg/m2	24.8 ± 2.6	26.2 ± 3.8	.07	24.8 ± 2.6	25.2 ± 3.1	.66

*Severity of coronary artery disease*						

Insignificant, *n* (%)	0 (0)	48 (100)		55 (100)	0 (0)	
1 VD, *n* (%)	38 (40.4)	0 (0)		0 (0)	13 (48.1)	
MVD, *n* (%)	56 (59.6)	0 (0)		0 (0)	14 (51.9)	

*Medical history*						

DM, *n* (%)	42 (44.7)	15 (31.2)	0.12	8 (14.5)	10 (37.0)	.02*
HTN, *n* (%)	73 (77.7)	29 (60.4)	0.03*	27 (49.0)	20 (74.1)	.03*
Dyslipidemia, *n* (%)	44 (46.8)	23 (47.9)	0.90	22 (40.0)	12 (44.4)	.70
Smoker, *n* (%)	38 (40.4)	14 (29.1)	0.16	23 (41.8)	9 (33.3)	.72

*Laboratory results*						

Total-C, mg/dL	176.3 ± 37.2	177.1 ± 35.0	.77	177.2 ± 34.8	177.7 ± 41.9	.96
LDL-C, mg/dL	107.4 ± 32.9	107.9 ± 28.5	.64	105.3 ± 31.9	107.9 ± 36.3	.88
HDL-C, mg/dL	42.3 ± 10.2	46.0 ± 10.1	.43	46.1 ± 12.1	43.6 ± 10.6	.01*
*hs*-CRP, mg/dL	0.50 ± 1.03	0.35 ± 0.57	.39	0.20 ± 0.32	0.38 ± 0.44	.73
CCr, mL/min	66.7 ± 16.8	76.3 ± 21.0	.47	71.3 ± 16.5	67.2 ± 15.1	.01
BNP, pg/mL^¶^	38.5	19.0	.01*	22.0	39.0	.002**
	(19.0–79.8)	(9.3–35.8)		(15.0–43.0)	(23.0–77.0)	

*Echocardiographic data*						

LV EF, %	63 ± 5	62± 6	.49	62 ± 6	64 ± 6	.54
LA, mm	39.1 ± 4.7	38.4 ± 4.9	.80	39.3 ± 4.4	39.0 ± 4.2	.90
*E*, m/s	0.60 ± 0.15	0.60 ± 0.15	.31	0.59 ± 0.16	0.64 ± 0.17	.28
*A*, m/s	0.76 ± 0.18	0.70 ± 0.18	.36	0.70 ± 0.22	0.76 ± 0.27	.30
*E/A* ratio	0.83 ± 0.27	0.89 ± 0.33	.35	0.90 ± 0.31	0.92 ± 0.31	.43
DT, ms	224.4 ± 61.9	214.1 ± 44.0	.85	232.0 ± 61.2	241.8 ± 44.7	.78

*SPECT data*						

LV EF, %	65.2 ± 7.1	66.0 ± 7.9	.69	67.8 ± 7.9	66.1 ± 6.7	.71
LVEDV, mL	85.7 ± 25.4	82.1 ± 20.4	.46	83.8 ± 23.0	82.9 ± 24.0	.12
LVESV, mL	30.9 ± 13.8	28.7 ± 11.5	.48	28.0 ± 12.4	28.8 ± 12.0	.49
SRS	1.032 ± 2.72	0.44±0.92	.46	0.22 ± 0.65	0.18 ± 0.73	.47
SSS	8.47 ± 5.61	2.53 ± 2.53	<.001***	2.37 ± 3.01	2.15 ± 2.31	.45
SDS	7.42 ± 4.64	3.27 ± 2.52	<.001***	2.15 ± 2.78	1.97 ± 2.08	.47

*P < .05; **P < .01; ***P < .001.

TP: true positive; FP: false positive; TN: true negative; FN: false negative; BMI: Body mass index, calculated as weight (kg) divided by square height (m^2^); VD: vessel disease; MVD: multi-vessel disease; Total C: total cholesterol; LDL-C: Low-density lipoprotein Cholesterol; HDL-C: High density lipoprotein Cholesterol; CCr: Creatinine clearance rate in milliliters per minute was calculated from the Cockcroft and Gault equation: ((140-age) × weight (kg)/serum creatinine (mmol/L)), multiplied by a constant of 1.25 in men and 1.03 in women; LV EF: Left ventricular Ejection fraction; LA: Left atrium; *E*: mitral peak early diastolic velocity; *A*: mitral peak late diastolic velocity; DT: *E* -wave deceleration time; LVEDV: left ventricular end-diastolic volume; LVESV: left ventricular end-systolic volume; SSS: summed stress score; SRS: summed rest score; SDS: summed difference score.

^†^Current or ex-smoker.

^¶^expressed as median (interpercentile).

**Table:** (a) Overall patients (*n* = 224).

	*r*	*P*
*Clinical*		

Age	0.367	<.001***
Creatinine clearance	−0.226	.001**
BMI	−0.010	.883

*Echocardiography*		

*E* wave	0.005	.95
*A* wave	0.194	.008**
Deceleration time	0.019	.801
LA size	0.089	.226
LVEF	0.019	.80
*E/A* ratio	−0.182	.014*

*SPECT*		

LVEF	−0.028	.681
LVESV	0.074	.271
LVEDV	0.064	.35
Summed rest score	0.249	<.001***
Summed stress score	0.179	.008**
Summed difference score	0.106	.12

**Table:** (b) Patients with true positive SPECT (*n* = 142).

	*r*	*P*
*Clinical*		

Age	0.393	<.001***
Creatinine clearance	−0.274	.001**
BMI	−0.018	.831

*Echocardiography*		

*E* wave	0.045	.65
*A* wave	0.339	<.001***
Deceleration time	0.032	.741
LA size	0.108	.249
LVEF	0.043	.649
*E/A* ratio	−0.183	.052

*SPECT*		

LVEF	−0.042	.619
LVESV	0.076	.372
LVEDV	0.099	.244
Summed rest score	0.309	<.001***
Summed stress score	0.233	.006**
Summed difference score	0.172	.043*

*P < .05; **P < .01; ***P < .001.

BMI: Body mass index *E*: mitral peak early diastolic velocity; *A*: mitral peak late diastolic velocity; LVEF: left ventricular ejection fraction; LVEDV: left ventricular end-diastolic volume; LVESV: left ventricular end-systolic volume.

**Table:** (a) Overall patients (*n* = 224).

	OR	95% CI	*P*
Age	1.005	0.965–1.046	.82
Diabetes mellitus	2.49	1.328–4.683	.004**
Hypertension	2.41	1.29–4.478	.025*
Creatinine clearance	0.99	0.97–1.006	.48
BNP ≥ 23.0 pg/mL	2.75	1.50–5.023	.001**

**Table:** (b) Patients with positive SPECT (*n* = 142).

	OR	95% CI	*P*
Age	1.00	0.94–1.05	.878
Diabetes mellitus	1.88	0.844–4.174	.122
Hypertension	2.03	0.88–4.63	.09
Creatinine clearance	0.98	0.95–1.00	.081
BNP≥23.0 pg/mL	3.34	1.51–7.37	.003**

*P < .05; **P < .01; ***P < .001.

**Table 4:** Diagnostic performance of BNP and SPECT for coronary artery disease.

	Sensitivity, %	Specificity, %	PPV, %	NPV, %
*Predicting CAD in overall patients (* *n* = 224)				

SPECT (+)	77.7	41.3	66.2	67.1
BNP ≥ 23.0 pg/mL	70.2	56.3	65.4	61.7

*Predicting CAD in patients with positive SPECT (* *n* = 142)				

BNP ≥ 23.0 pg/mL	68.1	62.5	78.0	50.0

*Predicting CAD in patients with negative SPECT (* *n* = 82)				

BNP ≥ 23.0 pg/mL	70.4	54.5	43.2	78.9
BNP ≥ 23.0 pg/mL	51.9	74.5	50.0	75.9

*Predicting CAD in patients with ischemia on LAD territory (* *n* = 59)				

BNP ≥ 23.0 pg/mL	73.7	57.1	75.7	54.5
BNP ≥ 23.0 pg/mL	64.9	59.0	72.7	50.0

*Predicting CAD in patients with ischemia on RCA or LCx territory (* *n* = 83)				

BNP ≥ 23.0 pg/mL	71.9	65.4	82.0	51.5
BNP ≥ 23.0 pg/mL	66.6	69.2	82.6	48.6

PPV: positive predictive value; NPV: negative predictive value.
